# Lean mass mediates the relation between temporal summation of pain and sex in young healthy adults

**DOI:** 10.1186/s13293-018-0200-z

**Published:** 2018-09-15

**Authors:** Abdulaziz Awali, Ali M. Alsouhibani, Marie Hoeger Bement

**Affiliations:** Clinical and Translational Rehabilitation Health Sciences Program, Department of Physical Therapy, College of Health Sciences, Marquette University, Milwaukee, WI USA

**Keywords:** Temporal summation, Lean mass, Physical activity, Body composition, Sex differences

## Abstract

**Background:**

Previous studies have shown that women experience greater temporal summation (TS) of pain than men using a repetitive thermal stimulus. These studies, however, did not individualize the thermal stimulus to each subject’s thermal pain sensitivity. The aim of this study was to investigate sex differences in TS using an individualized protocol and potential mediators that have been shown to influence TS including physical activity and body composition.

**Methods:**

Fifty young healthy men and women (21 men) participated in the study. Subjects completed TS testing on the right forearm using a repetitive thermal stimulus at a temperature that the subject reported 6/10 pain. Other testing included body composition (lunar iDXA), activity monitoring (Actigraph), and Pain Catastrophizing Scale (PCS).

**Results:**

Women reported greater TS than men (*p* = 0.019), and TS was correlated with right arm lean mass (*r* = − 0.36, *p* = 0.01) and magnification subscale of PCS (*r* = − 0.32, *p* = 0.03). Mediation analysis showed a complete mediation for the relation between sex and TS by right arm lean mass (indirect effect = 2.33, 95% BCa CI [0.42, 4.58]) after controlling for the temperature, the magnification subscale of PCS, and the average time spent in moderate to vigorous physical activity.

**Conclusion:**

The results of this study suggest that lean mass is a contributing factor to the sex differences in TS. Future studies should investigate whether interventions that increase lean mass have a positive effect on TS.

## Background

Temporal summation (TS) of pain is the increase in perceived pain in response to a tonic or repetitive noxious stimulus [15, 32] and may be used to assess central sensitization of pain. In pain-free individuals, some studies have shown sex differences in TS using a thermal stimulus with women experiencing greater TS than men [10, 20, 32, 35], while others reported no sex differences [1, 45]. The studies that showed sex differences in TS used the same temperature for both men and women without taking into consideration the potential sex differences in heat pain thresholds. For example, when using a repetitive fixed temperature protocol, women had greater TS and lower pain thresholds than men [10]. Thus, sex differences in pain threshold may contribute to differences in TS of heat between men and women. Individualizing the temperature of the thermal stimulus to the same pain intensity (e.g., 6/10 pain) rather than using the same temperature for all the participants would help to control for potential differences in pain threshold.

Understanding how men and women differ in endogenous pain modulation is complex but may include differences in physical activity and body composition. Vigorous activity, in particular, was a significant predictor for TS [28] while moderate and vigorous physical activity was negatively associated with pain intensity and unpleasantness in response to a thermal stimulus [8]. Physical activity may also prevent the development of chronic pain. In a longitudinal population-based study, individuals who reported at least moderate levels of exercise at baseline reported less pain over 12 months than individuals that reported no or light exercise [23]. A recent report from the Centers for Disease Control and Prevention showed men are more likely to meet the minimum weekly minutes required of moderate to vigorous physical activity than women [47]. Therefore, physical activity might be one of the contributing factors to the sex differences in TS.

Similarly, there are sex differences in body composition. In healthy young adults, men have greater lean mass and lower fat mass compared to women with the same body mass index (BMI) [49]. We have recently demonstrated that in normal weight and overweight/obese adolescents, regional lean mass (i.e., non-fatty or bony tissues) [18] predicted conditioned pain modulation (CPM) [40] and was positively associated with modulation of pain threshold following acute aerobic exercise [39]. Furthermore, obese individuals have greater heat pain threshold and tolerance in the abdominal region than individuals with normal weight while no differences were found in these measures when performed in an area with little fat like the forehead [33]. Another study has shown that obese individuals have similar heat pain thresholds to non-obese individuals, and heat pain thresholds were lower at the waist compared with the thenar eminence in both groups [44]. These studies show that regional fat or lean mass may influence measures of endogenous pain modulation.

The first aim of this study was to examine sex difference in TS using a repetitive individualized thermal stimulus in young healthy men and women. The second aim was to investigate potential mediators for the sex difference in TS including physical activity and body composition. We hypothesized the following: (1) women would report greater TS than men, and (2) the sex differences in TS would be mediated by physical activity and body composition.

## Methods

### Subjects

Fifty healthy individuals (21.59 ± 2.3 years old; 29 women) participated in the study. Subjects were recruited through flyers posted at Marquette University (Milwaukee, WI). All subjects were screened through the phone and excluded if they had an acute or chronic pain condition, cardiovascular problem, neurological disorder, limitations on participation in physical activity, mental health disorders, diabetes, or they were using medications that affect pain perception. Informed consent was obtained from all subjects. The institutional review board at Marquette University approved the protocol of this study.

### Protocol

Subjects participated in two randomized research sessions. During the first session, subjects completed the Pain Catastrophizing Scale (PCS), the trait version of the State-Trait Anxiety Inventory (STAI) and issued an Actigraph to wear for 7 days. During the second session, handgrip strength was measured. Body composition scan, 6-min walk, the state version of the STAI, and TS were randomized to either session and performed in this order with 10 min of rest between the 6-min walk test and TS test. During both sessions, two experimenters (one male and one female) were present and collected the data. This study was part of a larger study investigating endogenous pain modulation and body composition.

### Temporal summation of pain

Medoc Neurosensory Analyzer (TSA-II, Ramat Yishai, Israel) was used to deliver heat stimuli via a thermode to the volar aspect of the right forearm. Heat stimuli were individualized for each subject. The individualization task involved application of ten heat stimuli of random temperature that ranges from 40–49 °C with a baseline temperature of 39 °C. Subjects rated every stimulus using an 11-point numerical rating scale from 0 (no pain) to 10 (worst pain). The temperature that resulted in a pain rating of 6 (pain6) was used for the TS task [15]. If the subjects did not provide a pain rating of 6 (*n* = 19), then the highest temperature (49 °C) was used. Next, subjects received ten heat stimuli of the same intensity (pain6) with a ramp rate of 8 °C/s, 0.8 s at peak stimulus, and a return rate of 8 °C/s to the baseline temperature of 39 °C. The inter-stimulus interval was 1–1.1 s. Subjects were verbally cued during the test to rate the first, fifth, and tenth heat stimulus. The magnitude of TS was defined as the difference between the first and tenth pain ratings (tenth pain rating − first pain rating) [15]. The individualized TS protocol has been shown to have excellent reliability [21].

### Body composition

Lunar iDXA (GE, Madison, WI) was used for body composition quantification. Subjects were instructed to refrain from food and drink 1–2 h before the session and to remove all metal objects prior to the body scan. Scans were analyzed via Encore Software (GE, Madison, WI), and the obtained results included body mass index (BMI), total fat percentage, total lean mass (kg), Android/ Gynoid ratio (A/G ratio), right arm lean mass (kg), right arm lean percentage (%), right arm fat mass (kg), and right arm fat percentage (%). Because TS was applied over the right arm, we used the data from the arm for fat and lean mass as an indicator for the regional body composition.

### Physical activity and strength

#### Physical activity

Subjects wore an Actigraph (wGT3X-BT or wActisleep-BT, Pensacola, FL) on the non-dominant wrist for 7 days to quantify their physical activities. Subjects were instructed to complete daily logs regarding sleep time, physical activity, and any removal time. Data were downloaded and analyzed via Actilife software (Actilife 6.13.1, Pensacola, FL). Troiano algorithm and the daily logs were used to identify and remove the non-wear and sleep time from daytime physical activity calculation. The data of four valid days (wear time of at least 10 waking hours) were used for all subjects, which have been shown to be a representative for the data of 1 week [26]. Activities were divided into either sedentary/light activities or moderate to vigorous physical activities (MVPA) based on Freedson criteria [11, 12, 36].

#### Six-minute walk test

Subjects were asked to walk as fast as they can for 6 min. The test was performed on a straight 30-meter walking course in which the start and end points were marked with cones [22]. Standardized encouragement was provided for all subjects. Subjects were asked to rate their pain from 0 (no pain) to 10 (worst pain) [9] and to rate their rate of perceived exertion (RPE) from 0 (nothing at all) to 10 (extremely strong) [3]. Ratings for pain and RPE were provided at the beginning, middle, and the end of the test. The distance covered was measured.

#### Handgrip strength

A JAMAR Hand dynamometer (Lafayette, IN) was used to assess grip strength of the right hand. Subjects performed three brief maximum contractions (each lasted for 3–5 s) with encouragement. The highest value was used in the statistical analysis.

### Psychological outcomes

#### Pain Catastrophizing Scale

The PCS consists of 13 items that evaluate the exaggerated negative mental set towards pain [30, 41]. It has three main subscales, which are magnification (e.g., I become afraid that the pain will get worse), helplessness (e.g., I feel I cannot stand it anymore), and rumination (e.g., I cannot seem to keep it out of my mind). Subjects were instructed to complete the questionnaire in reference to past painful events and to rate each item on a Likert scale that ranges from 0 (not at all) to 4 (all the time). Total score and the score for each of the three subscales were calculated. Higher PCS scores indicate greater catastrophizing levels.

#### Anxiety

Subjects completed the STAI. The state version consists of 20 items in reference to how the subject feels at the moment. The trait version also has 20 items and refers to how the subject generally feels. Higher scores in STAI indicate greater anxiety levels [38].

### Statistical analysis

Data were analyzed using the Statistical Package for the Social Sciences (version 24, IBM, Armonk, NY). Normality and linearity were evaluated with Shapiro-Wilk test and visual inspection via Q-Q plots [14]. Extreme outliers with *z*-scores greater than 3.29 were detected and replaced with the next highest scores (Winsorization technique) [43]. Independent *t* test or Mann-Whitney *U* test were conducted for comparisons between men and women. Repeated measures analysis of variance (RM ANOVA) for the TS was done with a between-group variable (sex) and a within-group variable (stimulus number—first, fifth, and tenth heat stimulus), and the temperature of TS was used as a covariate. Independent *t* tests with Bonferroni correction were performed following the RM ANOVA in case of significant main effects or interaction. Pearson correlation was used to study the relation between TS and continuous variables. Point-biserial correlation was done to study the relation between sex and continuous variables. In case of non-normally distributed data, Spearman rank correlation was used. A *p* value of ≤ 0.05 was used. Data are presented in tables as mean ± standard deviations and in figures as mean ± standard error of the mean (SEM).

To determine the mediators of the relation between TS and sex, Sobel test was conducted using PROCESS in SPSS [16], and the variables that correlated were tested as mediators. For the mediation (indirect effect) to be significant, the bias-corrected confidence interval (95% BCa CI) should not include zero.

## Results

One outlier in each of the following variables was replaced with the closet score: BMI, right arm fat mass, and individualized temperature. The statistical analysis was performed with and without replacing the outliers, and there was no difference between the two approaches. There were two missing data points from the PCS caused by two subjects not answering one item. Six subjects were excluded from the Actigraph analysis caused by not meeting the criteria of wearing the Actigraph for at least 4 days (three subjects) and technical errors with the device resulting in no data collection (three subjects).

### Temporal summation

**Fig. 1:**
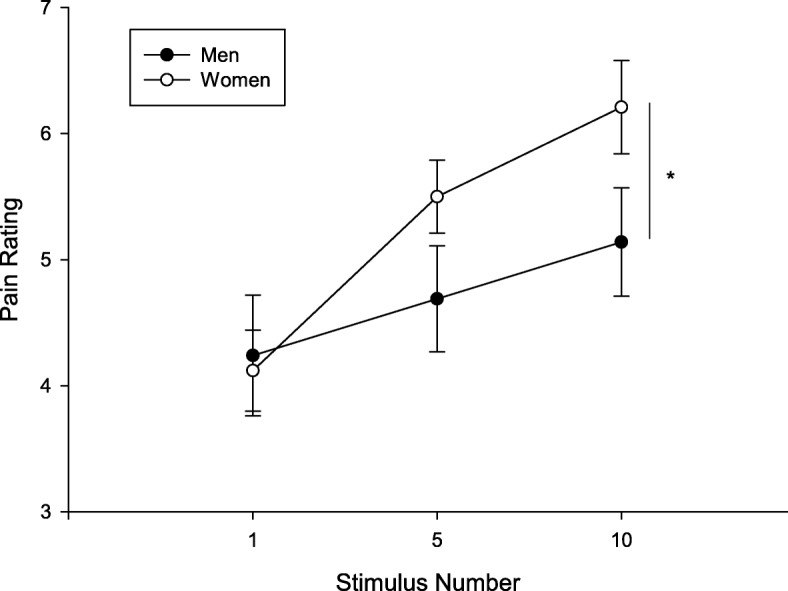
Women reported greater TS than men (main effect of stimulus number (*p* < 0.001); sex X stimulus number interaction *p* = 0.019). The data are presented as mean ± SEM. “*” indicates significant sex X stimulus number interaction

There was no significant difference between the individualized temperature that men and women reported as pain6 (Table 1). Nineteen participants (7 men and 12 women) did not report pain6; thus, the highest temperature (49 °C) was used as pain6 for the subsequent TS protocol. The magnitude of TS was significantly greater in women. RM ANOVA showed a significant main effect of stimulus number (*F* (1.35, 64.78) = 29.4, *p* < 0.001, *η*_*p*_^2^ = 0.380) and a significant sex X stimulus number interaction (*F* (1.35, 64.78) = 4.998, *p* = 0.019, *η*_*p*_^2^ = 0.094) (Fig. 1). After controlling for the effect of temperature on TS magnitude, the significance sex X stimulus number interaction remained (*F* (1.38, 64.65) = 4.76, *p* = 0.02, *η*_*p*_^2^ = 0.092). Post hoc test showed a difference between men and women in pain ratings for the tenth stimulus; however, this difference failed to reach the statistical significance level (*t* (48) = 1.89, *p* = 0.065). Point-biserial correlation showed a significant relation between sex and TS (*r* = 0.33, *p* = 0.02), which indicated that women tended to have greater TS than men. Pearson correlation showed a significant association between the individualized temperature and TS score (*r* = 0.38, *p* = 0.006); those with higher individualized temperatures had greater TS.

**Table 1:** Sex differences in TS, body composition, physical activity, and psychological outcomes

	Men (*n* = 21)	Women (*n* = 29)
	Mean ± SD	Mean ± SD
Age	22.1 ± 3.07	21.2 ± 1.49
TS		
TS temperature (°C)	48.05 ± 1.43	48.20 ± 1.37
TS heat magnitude	0.90 ± 1.58*	2.09 ± 1.87
Body composition (iDXA)		
BMI	25.28 ± 3.20**	22.48 ± 2.53
Total fat percentage (%)	22.23 ± 9.22**	30.38 ± 5.09
A/G ratio	0.95 ± 0.22**	0.78 ± 0.16
Total lean mass (kg)	60 ± 8.22**	39.93 ± 4.82
Right arm lean percentage (%)	77.24 ± 8.06**	64.55 ± 4.95
Right arm fat percentage (%)	19.05 ± 8.71**	32.14 ± 5.42
Right arm lean mass (kg)	4.22 ± 0.83**	2.10 ± 0.25
Right arm fat mass (kg)	1.01 ± 0.56	1.02 ± 0.28
Right handgrip strength (kg)	46.10 ± 8.47**	29.79 ± 4.67
Six-min walk test		
Covered distance (m)	639.4 ± 57.06	650.3 ± 36.86
Pain rating (at the middle)	0.5 ± 1.12	0.48 ± 0.95
RPE (at the middle)	2.64 ± 1.40	2.35 ± 1.38
Pain rating (at the end)	1.5 ± 2.15	0.83 ± 1.36
RPE (at the end)	3.5 ± 1.82	3.12 ± 1.50
Physical activity		
Daily average time in sedentary and light activities (min)	668.89 ± 73.96	707.68 ± 77.58
Daily average time in MPVA activities (min)	179.77 ± 68.30	186.24 ± 57.42
Average time in MVPA bout (min)	18.37 ± 3.02	16.64 ± 2.66
Daily average number of MVPA bouts	8.26 ± 3.28	9.02 ± 2.98
PCS		
Total PCS	13.43 ± 9.3	12.64 ± 8.97
Helplessness subscale	4.9 ± 4.3	4.5 ± 4.15
Magnification subscale	5.48 ± 4.08	4.79 ± 3.40
Rumination subscale	3.05 ± 2.03	3.36 ± 2.25
STAI		
Trait anxiety	33.52 ± 7.36	33.83 ± 8.41
State anxiety	25.10 ± 5.03	27.04 ± 6.11

*TS* temporal summation, *BMI* body mass index, *A/G ratio* Android/ Gynoid ratio, *RPE* rate of perceived exertion, *MVPA* moderate to vigorous physical activity, *min* minutes, *PCS* pain catastrophizing scale, *STAI* the State-Trait Anxiety Inventory. Data are presented as mean ± SD

*p < 0.05, **p < 0.01

### Body composition

**Fig. 2:**
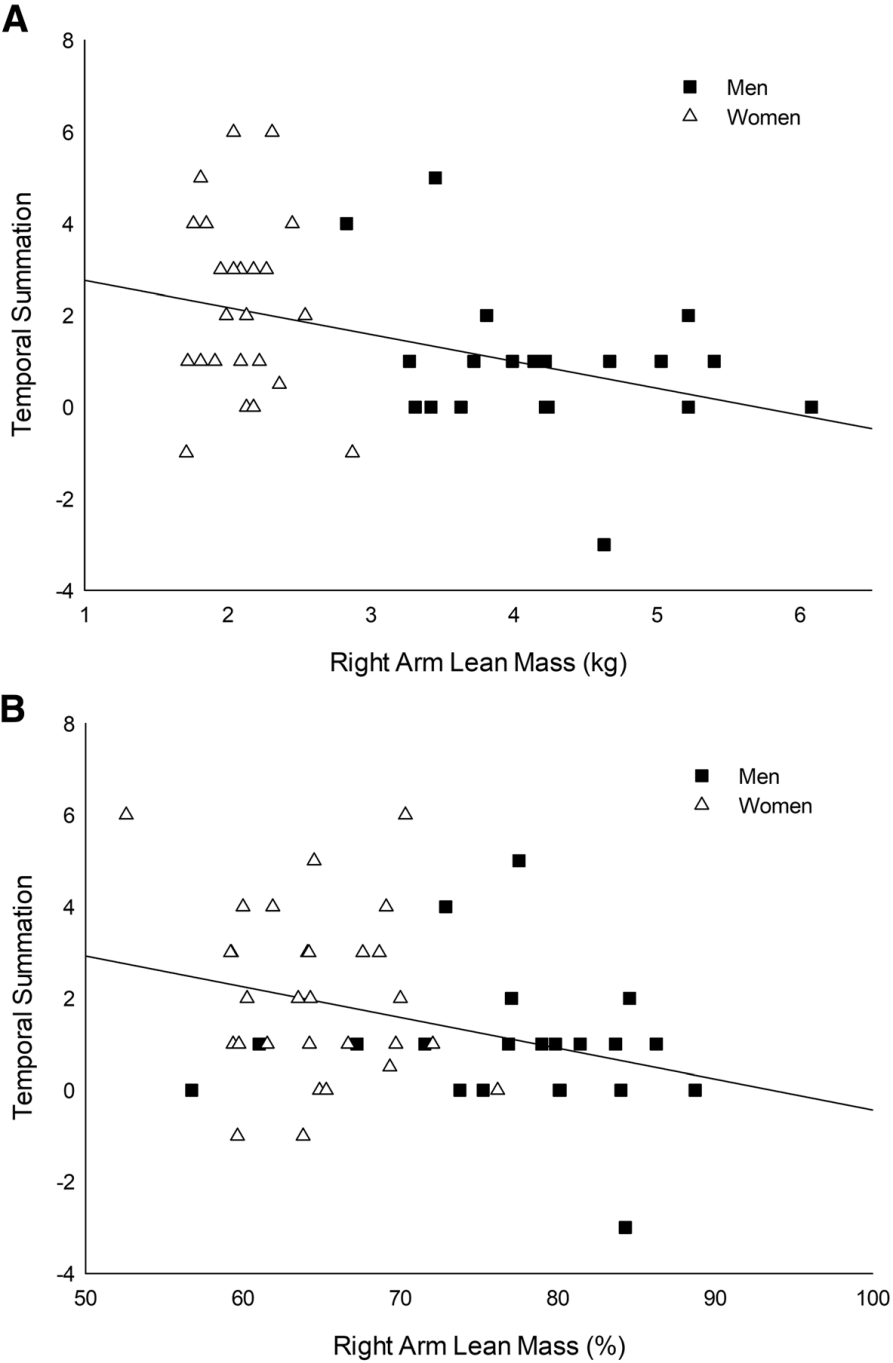
The correlation of TS and right arm lean mass and lean percentage. Adults with higher right lean mass [kg. (**a**) or % (**b**)] exhibit lower TS (*r* = − 0.36, *p* = 0.01; *r* = − 0.28, *p* = 0.04, respectively)

Men had greater BMI, greater A/G ratio, less total fat percentage, and more total body lean mass compared with women (Table 1). Specific to the right arm body composition, men had lower fat percentage (*p* < 0.01) and greater lean percentage (*p* < 0.01) than women. Right arm fat mass was not different between men and women; however, the right arm lean mass was greater in men (*p* < 0.01) (Table 1). The magnitude of TS was only correlated with the right arm body composition; there were negative correlations between TS and right arm lean mass (*r* = − 0.36, *p* = 0.01) (Fig. 2a) and right arm lean percentage (*r* = − 0.28, *p* = 0.04) (Fig. 2b). When the correlation analysis was conducted separately for men and women, there were no significant correlations.

### Physical activity and strength

There was no difference between men and women in the time spent in sedentary/light or MVPA activities (Table 1). Regarding the 6-min walk test, there was no difference in the distance covered, pain, and RPE at the middle or the end of the test. Men had significantly greater handgrip strength (*p* < 0.01). TS was not correlated with strength or physical activity variables when men and women were combined or separated.

### Psychological outcomes

**Fig. 3:**
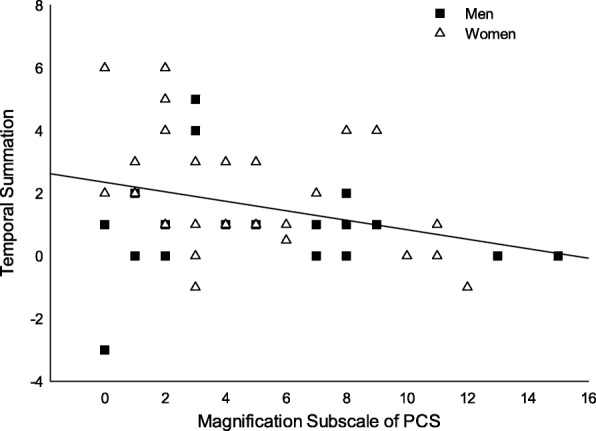
The correlation of TS and magnification subscale of PCS. Adults with higher magnification scores of PCS experience lower TS (*r* = − 0.32, *p* = 0.03)

Men and women had similar total PCS, subscales of PCS, and the state and trait anxiety questionnaires (Table 1). TS was negatively correlated with the magnification subscale of PCS (*r* = − 0.32, *p* = 0.03) (Fig. 3). No correlation was found with the other psychological outcomes. When the correlations were conducted separately for men and women, the magnification subscale of PCS was correlated with TS (*r* = − 0.43, *p* = 0.02) and the individualized temperature (*r* = − 0.40, *p* = 0.034) in women only.

### Mediation analysis

**Fig. 4:**
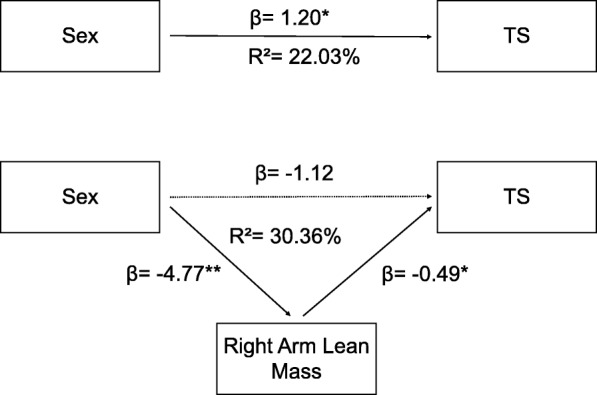
Mediation analysis for the relation between TS and sex. Right arm lean mass was the only mediator after adjusting for the temperature, magnification subscale of PCS, and MVPA (indirect effect = 2.33, 95% BCa CI [0.42, 4.58]). *p < 0.05; **p < 0.01; *β* = standardized coefficients

The mediation analysis was done to determine the mediators of the relation between TS and sex. The outcome variable was TS, and the independent variable was sex. The temperature used in the TS protocol and the magnification subscale of PCS were used as covariates because they were associated with the magnitude of TS. Right arm lean mass (indirect effect = 2.19, 95% BCa CI [0.68, 3.96]) was the only significant mediator for the relation between TS and sex. The mediation model also showed that the direct relation between sex and TS was no longer significant (direct effect = −1.12 95% BCa CI [−3.25, 1.01]), indicating a complete mediation. The mediation analysis was repeated with the addition of the average time spent in MVPA as a covariate to determine if lean mass was dependent on physical activity. Regional lean mass was still a significant mediator (indirect effect = 2.33, 95% BCa CI [0.42, 4.58]) (Fig. 4). Thus, the relation between TS and sex is completely mediated by the right arm lean mass even after adjusting for the effect of temperature, physical activity, and the magnification subscale of PCS.

## Discussion

This study took a novel and innovative approach by exploring the potential contributing factors related to the sex differences in TS that included individualizing the temperature of the thermal stimulus during TS protocol and investigating the involvement of anthropometrics. We demonstrated that sex differences in TS occur even when the repetitive thermal stimulus was individualized to pain 6/10, although others have shown no sex differences when using an individualized tonic temperature [45]. The second finding is that the relation between sex and TS is mediated by the right arm lean mass after controlling for the temperature, magnification subscale of PCS, and physical activity. Moreover, fat mass was not related to the TS, which is consistent with our previous study that did not find any relation between regional fat mass and CPM [40]. Thus, these results provide evidence that factors impacting nociceptive input from the periphery, such as lean mass, are related to central pain facilitation and mediate the sex differences in TS.

Men and women did not differ in the temperature used in the TS test, and the temperature was tailored for all subjects to their pain 6/10. Despite similar temperature, women reported greater TS than men, which is consistent with some previous studies that did not use an individualized thermal stimulus [10, 20, 32, 35]. Our results show that sex differences in TS are apparent even when the temperature thresholds are similar between men and women. In contrast, Tousignant-Laflamme and colleagues used an individualized temperature protocol that was tonically applied and found that there were sex differences in the individualized temperature but not TS [45]. Consequently, sex differences in TS may vary based on TS protocol parameters.

One of the interesting findings in this study was the negative association between the magnification subscale of PCS and TS. This contradicts previous studies that showed a positive correlation between PCS and TS [7, 13, 48]. The discrepancy between our findings and other studies could be caused by multiple factors. For example, we used a TS protocol that was individualized to each participant’s pain sensitivity, while the other studies utilized a fixed protocol that uses the same temperature for all participants. We also found a negative correlation between the individualized temperature and the magnification subscale of PCS. Thus, individuals with higher magnification scores reported higher pain ratings for lower temperatures resulting in lower temperature used in the TS protocol and subsequently lower TS score. Finally, we found a positive correlation between the individualized temperature and the TS magnitude, which is consistent with some previous studies [15, 31]. Consequently, high magnification of PCS may have opposite effects depending on the TS parameter. When individualizing the noxious stimulus prior to TS, higher magnification could result in lower temperatures and less TS (negative relation), whereas positive associations have been shown between magnification and TS when using a fixed temperature.

Right arm lean mass was the only mediator for the relation between TS and sex, and the individualized temperature for the thermal stimulus was 48 °C for both groups. One explanation for the relation between right arm lean mass and TS could be the sex differences in nociceptor density. Lauria et al. showed that some body composition variables were inversely related to epidermal nerve fiber density; men and women with higher BMI had lower nerve fiber density [24]. Similarly, Selim et al. found that women had greater epidermal nerve fiber density but similar heat pain thresholds compared to men [37]. Thus, nerve fiber density may help explain why greater TS occurs when stimulating larger body areas, due to subsequent activation of a larger number of nociceptors, than smaller areas [29]. In our study, men had higher BMI and more lean mass than women, which might have resulted in lower nerve fiber density and consequently lower TS and similar heat pain thresholds.

Another explanation could be the involvement of peripheral opioids. Muscles are one of the sources for opioid release [6], and opioids can decrease TS [42]. Animal studies have shown that activation of opioid receptors peripherally results in greater anti-nociceptive effects in males compared with females [4, 19]. TS was correlated with the regional lean mass and not with the total lean mass suggesting more of a local or peripheral effect rather than systemic. Thus, men may have greater opioid release and subsequent decrease in TS.

The activation of the descending inhibitory pathways could also be an explanation for the current findings. We have previously shown that regional lean mass was positively associated with the magnitude of CPM [40]. A recent study showed that in pain-free individuals, subjects with low TS of heat had more activation in areas of descending inhibition during TS protocol than those with enhanced TS [5]. Therefore, those with greater regional lean mass may have greater activation of areas of descending inhibition and consequently lower TS. Further mechanistic studies are needed to explore the relation between TS and lean mass.

Previous research has shown that moderate/vigorous physical activity, measured objectively by an Actigraph, was negatively associated with TS when the temperature was fixed at 46 °C but not at 48 °C [27]. In contrast, our results using Actigraph and an individualized temperature for TS showed that men and women had similar MVPA levels, which were not related to TS. Whether there were differences in resistance training, which increases lean mass, is not known because physical activity monitors do not adequately capture this type of exercise [34]. Besides the differences in TS protocol, our study had young physically active participants compared with the previous study with older less active participants. Therefore, the relation between physical activity and TS may change based on the TS protocol and the characteristics of the sample.

The finding of this study has potential clinical translation. First, understanding the factors that are related to pain sensitivity in healthy men and women provides insight into how they respond to tissue trauma (i.e., acute pain). This could lead to more effective acute pain management for early intervention protocols that could reduce the prevalence of chronic pain [25]. Second, identifying potential factors for the sex differences in TS could translate to future research initiatives in patient populations. For example, sex differences that have been reported in patients with chronic pain may be related to the sex differences in lean mass; women with knee OA have greater TS [2] and lower lean mass [46] compared with men with knee OA. Finally, our results may provide a potential mechanism for how resistance exercises improve central pain modulation. For example, resistance exercise training for 12 weeks has been shown to decrease TS in patients with OA [17]. The increase in lean mass that occurs with resistance training could explain the reductions in TS. Future interventional studies that involve resistance exercises should consider evaluating the change in body composition and its relation with the change in TS.

## Conclusion

In summary, women reported greater TS than men when the temperature for the noxious stimulus was individualized for all subjects, and the sex difference in TS was mediated by regional arm lean mass after adjusting for the temperature, pain catastrophizing, and physical activity while fat mass and physical activity were not associated with TS. Future studies are needed to investigate TS in body regions that differ in lean mass, patient populations, and rehabilitation interventions that increase lean mass.
